# Sleep Habits and Disorders in School-Aged Children: A Cross-Sectional Study Based on Parental Questionnaires

**DOI:** 10.3390/children12040489

**Published:** 2025-04-10

**Authors:** Luca Mezzofranco, Ludovica Agostini, Ayoub Boutarbouche, Sofia Melato, Francesca Zalunardo, Anna Franco, Antonio Gracco

**Affiliations:** Department of Neurosciences, University of Padua, Via Giustiniani 2, 35122 Padova, Italy; ludovica.agostini@gmail.com (L.A.); ayoub.boutarbouche@studenti.unipd.it (A.B.); sofia.melato@studenti.unipd.it (S.M.); zalunardo.francesca@gmail.com (F.Z.); anna.dott.franco@gmail.com (A.F.); antonio.gracco@unipd.it (A.G.)

**Keywords:** pediatric sleep disorders, sleep disturbances, children

## Abstract

Sleep is a crucial physiological process for cognitive, emotional, and physical development during childhood. Despite its importance, a significant percentage of school-aged children experience sleep disturbances, which can impact academic performance and overall well-being. This cross-sectional study aims to investigate sleep habits and disorders in children aged 6–13 years, identifying issues such as difficulties falling asleep, frequent awakenings, and parasomnias, as well as their correlations with daytime consequences. **Methods:** A structured questionnaire, based on the Sleep Disturbance Scale for Children (SDSC), was administered to 100 parents of school-aged children. The sample included participants without diagnosed neurological disorders, neurodevelopmental disorders, or chronic illnesses interfering with sleep. The data were statistically analyzed to assess the frequency and severity of sleep disturbances and their correlations with daytime symptoms. **Results:** Although most children (44.1%) slept 8–9 h per night, 32.4% exhibited bedtime resistance, and 29.4% had difficulty falling asleep. Common sleep disturbances included occasional snoring (44.1%), bruxism (11.8%), morning fatigue (41.2%), and daytime sleepiness (15.2%). Additionally, 23.5% of the children experienced confusion upon waking. The analysis also revealed a correlation between sleep fragmentation and mood alterations or cognitive difficulties. **Conclusions:** The study confirms the high prevalence of sleep disorders in pediatric populations, emphasizing the need for routine screening during clinical check-ups. Educational interventions on sleep hygiene practices—such as reducing evening screen exposure—and school policies that align with pediatric circadian rhythms could mitigate negative effects. The lack of objective measures such as actigraphy and polysomnography is a limitation, highlighting the need for integrated approaches in future studies. A multidisciplinary approach is essential to optimizing sleep health and overall child development.

## 1. Introduction

Sleep is an essential physiological process for the survival of all living organisms within the animal kingdom. It is a state of physical and mental rest characterized by a slowdown in neurovegetative functions and a reversible disconnection of consciousness from environmental stimuli. This complex process involves the interaction of multiple components of the central and autonomic nervous systems. In humans, sleep accounts for approximately one-third of life, with the remaining two-thirds dedicated to wakefulness. Non-REM (NREM) sleep phases are fundamental for physical recovery and energy replenishment [1].

During childhood, sleep facilitates the maturation of the central nervous system and strengthens the synaptic connections formed during the day. This explains why children, with their heightened learning capacity, exhibit a higher percentage of REM sleep compared to adults.

NREM sleep plays a crucial role in neuronal reorganization, eliminating redundant or non-useful connections to prevent informational overload [2]. This balance is regulated by a circadian process controlled by the hypothalamic suprachiasmatic nucleus, which modulates hormone production, such as serotonin during the day and melatonin at night. Melatonin secretion begins with the reduction in ambient light and peaks during nighttime hours, not only facilitating sleep onset by reducing vigilance but also synchronizing physiological rhythms, such as thermoregulation and cortisol secretion, with the light–dark cycle [3].

The interaction between melatonin and adenosine is particularly critical during childhood, a phase in which sleep–wake cycles are still maturing. Disruptions to this regulation (e.g., evening screen exposure and irregular sleep schedules) can compromise not only sleep duration but also its architectural quality, affecting the clearance of neurotoxic metabolites like beta-amyloid, which is associated with cognitive decline and Alzheimer’s disease [4].

Chronic sleep deprivation is linked to long-term neurodegenerative diseases due to the accumulation of toxic metabolites. In the short term, it results in reduced intellectual performance, concentration difficulties, memory loss, excessive daytime sleepiness, and mood alterations. Sleep deprivation also disrupts the autonomic nervous system, affecting the regulation of ghrelin and leptin hormones, leading to increased BMI and a predisposition to obstructive sleep apnea. Additionally, reduced glucose tolerance and insulin sensitivity increase the risk of type 2 diabetes [5].

A persistent misalignment between biological rhythms and social obligations, known as social jetlag, can have significant consequences on children’s sleep patterns, affecting their emotional regulation and school performance [6]. Identifying and addressing these issues is crucial for preventing long-term health complications.

## 2. Materials and Methods

A structured questionnaire, based on the Sleep Disturbance Scale for Children (SDSC) [7], was administered to a sample of 100 parents of children aged 6 to 13 years. The questionnaire consists of 26 items designed to assess various dimensions of pediatric sleep disorders over the past six months using a 5-point Likert scale (1 = never, 5 = always).

### 2.1. Population Characteristics and Selection Criteria

The study involved a cross-sectional sample of 100 parents of school-aged children (6–13 years). To ensure sample homogeneity, strict inclusion criteria were applied: children had to be regularly enrolled in primary or lower secondary schools, with no prior diagnosis of neurological disorders, neurodevelopmental disorders (such as autism or ADHD), or chronic diseases that could potentially interfere with sleep (e.g., epilepsy or severe asthma). Additionally, parents were required to complete the questionnaire in full.

Exclusion criteria aimed to minimize confounding factors: children undergoing pharmacological treatment with drugs known to alter the sleep–wake cycle (such as psychostimulants or antihistamines) and those exposed to acute stressful events within the family in the six months preceding the study (e.g., bereavement or parental separation) were excluded.

### 2.2. Questionnaire Structure

The questions were organized into six main domains to cover the primary sleep disorders:

1. Disorders of Initiating and Maintaining Sleep (DIMS): assessment of difficulties falling asleep, frequent awakenings, and trouble resuming sleep.
2. Sleep-Disordered Breathing (SBD): exploration of snoring, apnea [8]., and breathing difficulties.
3. Disorders of Arousal (DA): evaluation of phenomena such as sleepwalking, nightmares, and confusional arousals.
4. Sleep–Wake Transition Disorders (SWTD): analysis of involuntary body movements, teeth grinding (bruxism), and abnormal behaviors during sleep onset.
5. Disorders of Excessive Somnolence (DOES): investigation of difficulties waking up, morning fatigue, excessive daytime sleepiness, and sudden sleep episodes. As highlighted in recent research, excessive daytime sleepiness and difficulties in sleep–wake regulation can significantly impact overall well-being and quality of life in affected individuals [6].
6. Sleep Hyperhidrosis (SHY): measurement of excessive sweating during sleep.

### 2.3. Data Collection Methods

The questionnaire was administered online, ensuring participant anonymity and privacy. Parents were asked to complete the questionnaire based on their observations of their children over the past six months.

### 2.4. Data Analysis

The collected data were statistically analyzed to carry out the following:

1. Compute specific scores for each sleep disorder category by summing the items associated with each dimension.
2. Determine the total SDSC score, indicative of the overall severity of sleep disorders.
3. Identify recurring patterns and analyze significant deviations from SDSC normative values.
4. Examine correlations between sleep dimensions and daytime consequences (e.g., fatigue, sleepiness).

## 3. Results

### Sample Characteristics

The sample consisted of a pediatric population with an equal gender distribution (50% male, 50% female). Regarding the age distribution, 18 children were under 5 years old, 27 were between 10 and 12 years old, and 55 were under 10 years old.

In terms of parental demographics, 17.6% were younger than 35 years, 50% were under 45 years, and 32.4% were under 55 years. Mothers accounted for 81.8% of respondents, while fathers represented 18.2%.

Regarding educational background, 82.4% of parents held a university degree or a higher education diploma, while 14.7% had completed secondary education.

The data collected through the questionnaire provided insights into various aspects of children’s sleep, including duration, quality, and associated difficulties (Table 1).

**Figure 1:**
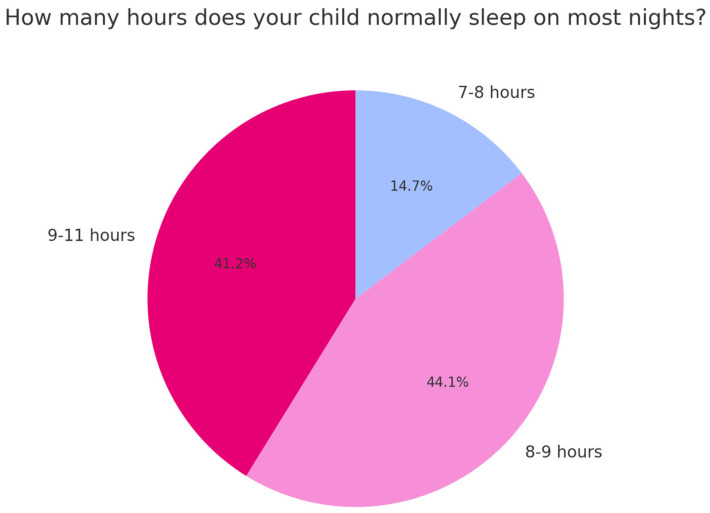
Time to fall asleep once in bed.

The majority of children (44.1%) reported sleeping between 8 and 9 h per night (Figure 1), followed by 41.2% who slept between 9 and 11 h. A smaller proportion (14.7%) slept between 7 and 8 h.

**Figure 2:**
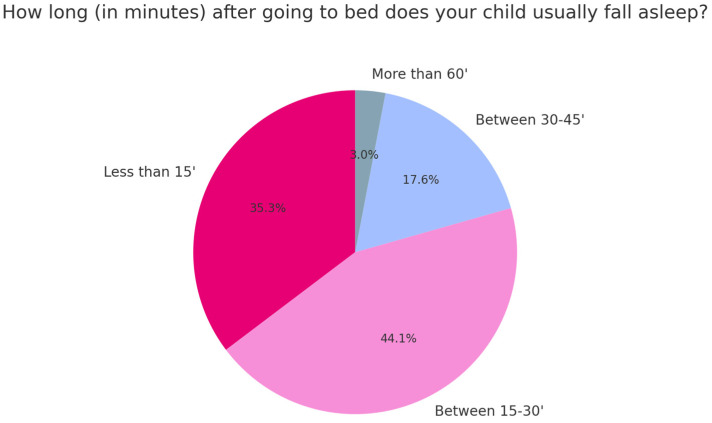
Time it takes children to fall asleep after going to bed.

Regarding sleep onset latency, a portion of the children (35.3%) fell asleep within 15 min, while 44.1% took between 15 and 30 min. A minority (3%) required more than 60 min to fall asleep (Figure 2).

Approximately 32.4% of children occasionally resisted going to bed, while another 32.4% exhibited this behavior one to two times per week. Only 14.7% showed a reluctance to go to bed daily.

Half of the parents (50%) reported occasional difficulties in their children’s sleep onset, while 29.4% observed such difficulties one to two times per week. Only 5.9% encountered frequent difficulties.

Half of the children (50%) did not exhibit signs of anxiety or fear during the sleep onset process, while 32.4% occasionally displayed mild anxiety. Similarly, 50% did not show involuntary movements during sleep, although 14.7% occasionally exhibited sudden jerks or movements. The majority of children (91.2%) did not engage in repetitive behaviors such as rocking or head banging during sleep. Regarding vivid dreams, 76.5% did not report experiencing intense dream episodes, whereas 20.5% experienced them occasionally.

Most children (64.7%) did not experience excessive sweating during sleep, although 8.8% reported frequent sweating, and 20.6% experienced it occasionally. About 47.1% of children did not wake up during the night, while 32.4% woke up occasionally (one to two times per month or less). Among those who experienced frequent awakenings, 5.9% did so three to five times per week. Nearly half of the children (47.1%) had no difficulty returning to sleep after waking up, whereas 38.3% faced occasional challenges. A smaller group (11.8%) reported frequent difficulty returning to sleep.

In terms of movement during sleep, 35.3% of children moved occasionally, 23.5% did not display such movements, and 11.8% moved frequently. Most children (79.4%) did not experience breathing difficulties during sleep, and 38.2% did not snore. However, 44.1% reported occasional snoring. Sleepwalking was absent in 88.2% of children, and 42.4% did not engage in sleep talking.

Bruxism was not reported by 58.8% of children, although 11.8% occasionally exhibited this behavior. Post-awakening behaviors indicated that 70.6% of children did not display episodes of disorientation or confused screaming, while 23.5% experienced such episodes occasionally.

While 38.2% of children did not wake up feeling tired, 41.2% occasionally felt fatigued. Daytime sleepiness was not an issue for 72.7% of children, with 15.2% reporting occasional episodes. Finally, most children (85.3%) did not fall asleep suddenly in inappropriate situations, although 14.7% reported occasional episodes of inappropriate sleep.

## 4. Discussion

The findings of this study highlight the multifaceted nature of pediatric sleep, revealing both adherence to recommended sleep duration and notable challenges in sleep quality and continuity. While most children met age-appropriate sleep requirements, the prevalence of difficulties in falling and staying asleep underscores this as an area of concern. These challenges parallel the occurrence of parasomnias, which, although often transient, can significantly impact children’s well-being and development if persistent.

The variability in sleep patterns underscores the importance of considering individual and environmental factors that influence sleep. For instance, cultural differences, parental routines, and exposure to electronic devices may play a crucial role in shaping children’s sleep behaviors. The observed relationship between excessive daytime sleepiness and fragmented nighttime sleep aligns with previous research, suggesting that early identification and intervention are critical to preventing further complications, such as academic difficulties and mood dysregulation [9].

The high prevalence of snoring and occasional respiratory disturbances among participants warrants attention. These findings may indicate underlying conditions, such as obstructive sleep apnea (OSA), which is associated with an increased risk of cardiovascular complications, including hypertension and heart rhythm abnormalities [10], as well as neurocognitive deficits, such as a reduced attention span and memory impairment [11]. Similarly, the frequent reports of bruxism and movement-related sleep disruptions raise questions about the potential role of stress, anxiety, or physiological factors in modulating these behaviors.

A critical element emerging from this investigation is the frequent resistance to bedtime (32.4% of children 1–2 times per week) and difficulty in morning awakenings (26.5% of cases). These behaviors may reflect an emerging social jetlag, where school schedules conflict with children’s natural circadian tendency to sleep longer in the morning. If prolonged, such misalignment is associated with reduced sleep duration, metabolic alterations, and impaired cognitive performance, suggesting the need for educational policies that delay school start times to better align with pediatric biological rhythms.

The presence of post-awakening confusion and fatigue in a substantial subgroup of children highlights the broader implications of inadequate sleep quality. These issues are not only disruptive for the child but also affect family dynamics, emphasizing the need for educational campaigns to promote better sleep hygiene. Strategies such as consistent bedtime routines, limiting screen time before sleep, and creating an optimal sleep environment can serve as preventive measures. Additionally, the involvement of healthcare providers, including pediatricians, sleep specialists, and mental health professionals, is essential for managing complex cases.

Future research should expand on these findings by incorporating objective sleep measurements, such as polysomnography or actigraphy, to validate parental reports and provide a deeper understanding of the physiological underpinnings of pediatric sleep disorders. Longitudinal studies examining the evolution of sleep patterns and their long-term effects on health outcomes are also needed to guide interventions.

### Study Limitations

Despite the use of a structured and validated tool, reliance on parent-reported data, while practical for large-scale screening, introduces potential recall and observation biases. For example, parents may underestimate nighttime awakenings or overinterpret typical behaviors as pathological. To mitigate these limitations, future studies should integrate objective measures such as actigraphy to quantify sleep–wake patterns or polysomnography to assess respiratory disturbances and sleep architecture. Combining subjective and objective data would enhance the validity of findings, particularly for conditions such as obstructive sleep apnea or periodic limb movement disorder.

The study did not directly investigate participants’ chronotype, which refers to individual preferences for morningness or eveningness—a factor that modulates vulnerability to social jetlag. Future research could incorporate tools such as the Morningness–Eveningness Questionnaire [12] to explore how the interaction between biological rhythms and social commitments influences sleep quality in pediatric populations.

## 5. Conclusions

This study provides a critical overview of sleep habits and disorders in school-aged children, highlighting significant variability between adherence to recommended sleep duration and challenges related to sleep quality and continuity. The collected data reveal that, despite most children obtaining an adequate number of sleep hours, a considerable proportion experiences difficulties in sleep onset, frequent awakenings, occasional snoring (44.1%), and behaviors such as bruxism (11.8%). These disturbances are associated with relevant daytime consequences, including fatigue (41.2%), post-awakening confusion (23.5%), and sleepiness (15.2%), with direct implications for academic performance and emotional well-being.

The findings emphasize the necessity of routine sleep screenings during pediatric check-ups, particularly for children exhibiting daytime symptoms such as attention difficulties or irritability. Interventions should include family education on sleep hygiene practices, with a specific focus on reducing blue light exposure from screens before bedtime, which is known to suppress melatonin synthesis and delay sleep onset [13].

Standardizing bedtime and wake-up schedules, even on weekends, is crucial to synchronizing circadian rhythms.

School policies that—where possible—delay the start of classes could help mitigate social jetlag, which arises from the misalignment between pediatric biological rhythms, naturally shifted toward later phases, and social commitments [6].

Finally, a strong multidisciplinary collaboration between pediatricians, sleep specialists, and psychologists is essential for managing complex cases, such as those with suspected obstructive sleep apnea (OSA) or psychiatric comorbidities.

While parental questionnaires provide useful data, future studies should integrate objective measures such as actigraphy and polysomnography to validate findings and further investigate physiological mechanisms, including beta-amyloid accumulation and glymphatic system activity. Additionally, incorporating assessments of children’s chronotypes using tools such as the Morningness–Eveningness Questionnaire [12] would allow researchers to explore how circadian preferences interact with daily commitments.

In conclusion, promoting quality sleep in childhood requires a holistic approach that combines prevention, education, and environmental adaptations, with the goal of safeguarding not only physical health but also the cognitive and emotional development of future generations.

## Figures and Tables

**Table 1:** Questionnaire results.

Question	Never (%)	Occasionally 1–2 Times Per Month (%)	Sometimes 1–2 Per Week (%)	Often (%)	Always, Every Day (%)
The child goes to bed unwillingly	14.7	32.4	32.4	20.6	14.7
The child has difficulty falling asleep	14.7	50	29.4	5.9	0
The child feels anxious or scared when falling asleep	50	32.4	8.8	8.8	0
The child jerks or makes sudden movements of certain parts of the body while falling asleep	50	29.4	14.7	5.9	0
The child shows repetitive actions such as rocking or banging his head while falling asleep	91.2	2.9	2.9	2.9	0
The child experiences vivid dream scenes as he falls asleep	76.5	20.5	2.9	0	0
The child sweats excessively while falling asleep	64.7	20.6	12.5	8.3	4.2
The child wakes up more than twice and during the night	47.1	32.4	14.7	0	0
If the child wakes up during the night, he has difficulty falling back asleep	47.1	38.3	11.8	0	0
The baby moves continuously during sleep	23.5	23.5	35.3	0	11.8
The child gasps or is unable to breathe during sleep	79.4	11.8	0	0	0
The child snores.	38.2	44.1	8.8	0	0
Baby sweats excessively during the night	47.1	32.4	11.8	8.8	0
Have you noticed episodes of sleepwalking in the child? (gets out of bed, walks)	88.2	11.8	0	0	0
Have you observed the baby talking in his sleep?	42.4	42.2	12.1	0	0
Child grinds teeth during sleep	58.8	11.8	20.6	8.8	0
The child wakes up screaming or confused in such a way that you can’t communicate with him but has no memory of these events the next morning.	70.6	23.5	0	0	0
The child has nightmares that he does not remember the next day.	35.3	55.9	0	0	0
The child has difficulty waking up in the morning.	29.4	32.4	26.5	0	0
The child wakes up feeling tired in the morning.	38.2	41.2	11.8	8.8	0
The child feels unable to move or feels paralyzed when he wakes up in the morning.	88.2	11.8	0	0	0
The child has daytime sleepiness.	72.7	15.2	12.1	0	0
The child suddenly falls asleep in inappropriate situations.	85.3	14.7	0	0	0

## Data Availability

The original contributions presented in the study are included in the article, further inquiries can be directed to the corresponding author.
